# Pulsatilla Saponin B4 Ameliorates LPS-Induced Inflammatory Response by Inhibiting IL-17RA and MAPK/NF-κB Signaling in Bovine Mammary Epithelial Cells and Mastitis Mouse Model

**DOI:** 10.3390/vetsci13060521

**Published:** 2026-05-27

**Authors:** Hao Zhang, Shouli Yi, Panpan Ding, Baocheng Hao, Yu Liu, Zhen Yang, Hongjuan Zhang, Di Wu, Dan Shao, Shengyi Wang

**Affiliations:** 1 Key Laboratory of Veterinary Pharmaceutical Development of Ministry of Agriculture, Lanzhou Institute of Husbandry and Pharmaceutical Sciences, Chinese Academy of Agricultural Sciences, Lanzhou 730050, China; zh1130334945@outlook.com (H.Z.); yishouli@st.gxu.edu.cn (S.Y.); 18419713523@163.com (P.D.); haobaocheng@caas.cn (B.H.); liuyu8108@163.com (Y.L.); lzmy_yz@163.com (Z.Y.); zhanghongjuan@caas.cn (H.Z.); wudi@caas.cn (D.W.); 2 College of Veterinary Medicine, Gansu Agricultural University, Lanzhou 730070, China; 3 College of Animal Science and Technology, Guangxi University, Nanning 530004, China; 4 College of Veterinary Medicine, Anhui Agricultural University, Hefei 230036, China

**Keywords:** Pulsatilla saponin B4, bovine mastitis, IL-17RA signaling, MAPK/NF-κB signaling

## Abstract

Bovine mastitis is a persistent and challenging illness in dairy industry, bringing about devastating economic losses to the sector. The longstanding over-reliance on antibiotic therapy has raised severe public health concerns, highlighting the critical need to develop safe and effective alternative regimens. Pulsatilla saponin B4 (PSB4), a plant-derived triterpenoid saponin, has been shown to regulate inflammation. However, the effect of PSB4 on mastitis in lactating cows has not been elucidated. The aim of this study was to explore the anti-inflammatory property and the molecular mechanisms of PSB4 in bovine mammary epithelial cells (BMECs) and mouse mastitis model. In cultured cells, PSB4 alleviated LPS-induced inflammatory reaction by suppressing the expression of pro-inflammatory mediators in BMECs. Notably, RNA-seq analysis indicated that the anti-inflammatory effect of PSB4 was correlated with IL-17RA and NF-κB signaling, with subsequent Western blot validation. Moreover, BMECs were stimulated with recombinant interleukin-17A (rIL-17A) to induce inflammation and challenged with Taltz to specifically inhibit IL-17RA signaling. The results showed that PSB4 reversed the rIL-17A-induced upregulation of elements within the IL-17RA pathway and its downstream MAPK/NF-κB cascade, including their downstream effectors. Furthermore, Taltz blocked the efficacy of PSB4 in protecting against LPS-induced inflammation. In vivo, PSB4 alleviated the inflammatory damage of mammary gland, pro-inflammatory mediator levels in mammary gland tissue and se-rum, while blocking the activation of IL-17RA signal along with downstream MAPK/NF-κB signal in LPS-induced mouse mastitis model. Taken together, studies in both BMECs and mice demonstrated that PSB4 alleviates mastitis by inhibiting IL-17RA signaling and downstream MAPK/NF-κB pathway, which may be a new strategy and a target for the management of mastitis.

## Simple Summary

Mastitis, an infection of the cow’s udder, is a serious problem for dairy farmers because it reduces milk quality and causes large financial losses. Currently, farmers often rely on antibiotics to treat this disease, but overuse of antibiotics can lead to drug-resistant bacteria, which threatens human health. Therefore, new, safer treatments are urgently needed. This study tested a natural compound called Pulsatilla saponin B4, which comes from a traditional Chinese herb known as Pulsatilla. Using cow udder cells in the laboratory and a mouse model of udder infection, the researchers found that this compound effectively reduced inflammation caused by bacterial toxins. Further experiments showed that the compound works by blocking a specific inflammatory pathway known as IL-17RA, which acts like a switch for inflammation. When this pathway was blocked, the compound could no longer work, confirming its key role. In both cell tests and living mice, the treatment lowered signs of infection and tissue damage. These findings suggest that this plant-derived compound could lead to a new, antibiotic-free treatment for mastitis, benefiting farmers by reducing losses and protecting public health by limiting antibiotic overuse.

## 1. Introduction

Bovine mastitis represents a common inflammatory condition in the dairy sector [1,2,3]. It not only impacts both milk production and composition but also results in significant financial losses for the dairy industry [4,5,6,7]. The control of bovine mastitis currently relies heavily on antibiotics. Yet overuse results in significant milk drug residues and increases antimicrobial resistance, compounding the difficulties in managing the disease. Notably, it also results in antibiotic residues in the dairy supply chain, representing a grave peril to human health. Therefore, the development of novel antibiotic alternatives is of critical significance.

The occurrence of bovine mastitis results from the failure of mammary gland defenses, allowing successful pathogen invasion and triggering excessive immune mediated tissue damage [8,9]. Over the past few years, extensive studies have shown that IL-17 signal transduction plays a crucial role in immune-inflammatory diseases [10,11]. IL-17 is a 155-residue glycoprotein. This cytokine family includes six members, from IL-17A through IL-17F. The IL-17R family comprises five distinct sub-units, ranging from IL-17RA to IL-17RE [12,13]. Within the IL-17 family, IL-17A serves as the primary effector while being the most extensively characterized pro-inflammatory cytokine [14]. Typically, upon binding to the IL-17RA receptor, IL-17A recruits the adaptor molecule ACT1. Acting as an E3 ubiquitin ligase, ACT1 then facilitates the ubiquitination of TNF receptor-associated factor 6 (TRAF6), thereby concurrently activating both the MAPK (p-ERK, p-p38 and p-JNK) and NF-κB (p-p65 and p-IκBα) signaling. The activation of these signaling subsequently drive the pro-duction of various inflammatory mediators, including pro-inflammatory cytokines (IL-6, TNF-α and IL-1β) and chemokines (CXCL1, CXCL2, CXCL5, CXCL8, CXCL10, CCL2 and CCL20), ultimately amplifying the inflammatory response [15]. Evidence suggests that certain Chinese herbal medicines and their extracts can alleviate inflammatory diseases by suppressing IL-17RA signaling. Liang et al. [16] found that gingerone A can suppress inflammation while repairing intestinal barrier function through targeting IL-17RA in ulcerative colitis. Gong et al. [17] demonstrated that the Danggui Beimu Kushen Pill alleviates colitis by suppressing inflammation through modulation of the IL-17A/IL-17RA pathway. Li et al. [18] demonstrated that isoliquiritigenin treats myofascial pain syndrome in rats through blockade of the IL-17RA/ACT1/p38 cascade, thereby reducing sarcomeric contraction and inflammation. The above results suggest that candidate drugs targeting IL-17RA signaling may be a strategy for treating mastitis in dairy cows.

PSB4, a triterpene saponin derived from the medicinal plant *Pulsatilla chinensis*, represents a natural bioactive compound. It possesses anti-inflammatory, antioxidant, antitumor, and immunomodulatory activities [19,20]. Research has demonstrated that PSB4 exerts a favorable therapeutic action against colitis [21,22,23,24,25]. Luo et al. [26] demonstrated that PSB4 ameliorates oral lesions in a rat model through regulation of intestinal microflora and its metabolites. Yuan et al. [27] demonstrated that PSB4 exerts a protective effect against acute lung damage through simultaneous suppression of NLRP3 inflammasome activation and TLR4 dimerization. The above research shows the efficacy of PSB4 in controlling inflammation and its strong potential for treating bovine mastitis, we speculated that PSB4 might have therapeutic effects on bovine mastitis. Accordingly, this work focused on the effects of PSB4 on LPS-triggered inflammation, using BMECs and a mouse mastitis model, along with its underlying mechanism. Our results demonstrated that PSB4 effectively attenuated LPS-induced inflammation in both BMECs and mice. We further confirmed that its anti-inflammatory effect is mediated by inhibiting IL-17RA signaling and downstream MAPK/NF-κB signaling. The results provide novel insights and potential therapeutic targets for the antibiotic-free treatment of bovine mastitis.

## 2. Materials and Methods

### 2.1. Regents

PSB4 standard (purity > 98%, 129741-57-7) was purchased from Beijing Solarbio (Beijing, China). LPS (L2880-100MG) was bought from Sigma Aldrich (St. Louis, MO, USA). Ixekizumab (Taltz, HY-P9924) was purchased from Med ChemExpress (Shanghai, China). Recombinant bovine IL-17A protein (CSB-YP724243BO) was purchased from cusabio (Wuhan, China).

Preparation of LPS and PSB4: For experiments with BMECs, LPS and PSB4 were dissolved in BMEC culture medium and diluted to the desired working concentrations immediately before use. For mouse experiments, LPS and PSB4 were dissolved in sterile normal saline and diluted to the required concentrations. The specific treatment regimens for each experiment, including drug concentrations, treatment duration, and sample collection time points, are described in the respective figure legends.

### 2.2. Mice and Treatments

BALB/c mice (2–3 months old, 30–35 g) were sourced from the Experimental An-imal Center of the Lanzhou Veterinary Research Institute, Chinese Academy of Agri-cultural Sciences. Animals were maintained under standard conditions (12 h light/dark cycle, 23–25 °C) with ad libitum access to food and water. After a 2-day ac-climatization period, the mice were individually caged. Daily monitoring of pregnant mice was conducted to record parturition timing. Following delivery, 36 dams were randomly allocated to three experimental groups (*n* = 12 per group): (1) Control group, (2) LPS group, and (3) LPS + PSB4 group. On lactation days 3, 5, and 7, mice from both the LPS and LPS + PSB4 groups received an intramammary dose of LPS (50 µL of a 0.2 mg/mL solution) into both fourth mammary glands. Starting from lactation day 3, the LPS + PSB4 group was administered PSB4 (50 mg/kg/day) once daily via intraperitoneal injection for 7 uninterrupted days. Control animals received an equal volume of saline by intraperitoneal injection daily from day 3, also for 7 consecutive days. On lactation day 9, all mice were euthanized, and blood and mammary gland tissue samples were harvested for subsequent analysis. For qRT-PCR analysis, mammary tissue samples from all 12 mice per group were used (*n* = 12). For protein extraction and Western blot analysis, 3 mice were randomly selected from each group (*n* = 3). All animals were handled in compliance with the guidelines of the Declaration of Helsinki and received approval from the Institutional Animal Care and Use Committee of the Lanzhou Institute of Husbandry and Pharmaceutical Sciences of the Chinese Academy of Agri-cultural Sciences (Approval No. 2025-23).

### 2.3. Cell Culture and Treatments

The BMECs used in this study are a commercially available low-passage (Passage 4) bovine mammary epithelial cell line, purchased from Jennio Biotechnology Co., Ltd. (Guangzhou, China). Cells were maintained in RPMI 1640 culture medium containing 10% fetal bovine serum (FBS), both from Gibco (Grand Island, NY, USA), and incubated at 37 °C in a humidified atmosphere with 5% CO_2_. Before digestion, the cells were rinsed with phosphate-buffered saline (PBS). After that, the cells were collected using 0.25% trypsin-EDTA (Gibco, Grand Island, NY, USA). After terminating digestion with RPMI 1640/10% FBS, cells were pelleted by centrifugation and resuspended in fresh medium for subculturing.

For treatments, BMECs were seeded into culture plates and allowed to adhere. In the LPS-induced inflammation model, cells were exposed to LPS (10 μg/mL) for 6 h, after which PSB4 was added at final concentrations of 25, 50, and 100 μg/mL without changing the culture medium, and incubation was continued for another 18 h. For recombinant IL-17A (rIL-17A) stimulation, cells were treated with rIL-17A (1 μg/mL) for 6 h, followed by incubation with PSB4 (100 μg/mL) for 18 h. In the IL-17RA blockade experiment, cells were first exposed to LPS (10 μg/mL) for 4 h, then incubated with the IL-17RA antagonist Taltz (ixekizumab, 10 μg/mL) for 2 h, and subsequently treated with PSB4 (100 μg/mL) for a further 18 h. At the end of each treatment, cells and culture supernatants were collected for downstream analyses. All in vitro experiments were performed with three independent biological replicates (*n* = 3), and each biological replicate included three technical replicates.

### 2.4. Cell Viability Assay

BMECs viability was assessed using the CCK-8 (NCM Biotech, Suzhou, China), performed as specified by the manufacturer.

### 2.5. Enzyme-Linked Immunosorbent Assay

The concentrations of IL-6, TNF-α, IL-1β and IL-17A in BMEC cultures were measured with commercial ELISA kits (all from MLBIO, Shanghai, China), following the manufacturers’ protocols. The respective catalog numbers were as follows: ml064296V for IL-6, ml077389V for TNF-α, ml064295V for IL-1β, and ml063477 for IL-17A.

### 2.6. Quantitative Real-Time PCR

Total RNA was isolated from the cells with TRNzol Universal Reagent (TIANGEN, Beijing, China) following the manufacturer’s instructions. RNA concentration and purity were measured with an Eppendorf BioPhotometer^®^ D30 (Eppendorf, Hamburg, Germany). cDNA was then synthesized using a 96-well thermal cycler (Applied Biosystems, Applied Biosystems, Thermo Fisher Scientific, Waltham, MA, USA) and the Evo M-MLV RT Premix Kit (Accurate Biotechnology, Changsha, China). qPCR was conducted on an Applied Biosystems real-time PCR instrument (Foster City, CA, USA). All primer pairs were designed by the authors, and their amplification efficiencies were validated in preliminary experiments by standard curve analysis using serially diluted pooled cDNA. Gene expression levels were normalized to β-actin and quantified using the 2^−ΔΔCt^ method. Primer sequences for bovine and murine genes are listed in Table 1 and Table 2, respectively.

### 2.7. Western Blotting

BMECs and mouse mammary tissue were lysed in RIPA buffer with inhibitors (Beyotime, Shanghai, China). Protein concentrations were determined using a BCA kit (Beyotime). Equal amounts of protein were separated by SDS-PAGE and transferred onto PVDF membranes (Merck, Darmstadt, Germany). Membranes were blocked with 5% skim milk (2 h, RT), incubated with primary antibodies (4 °C, overnight), followed by HRP-conjugated secondary antibodies (3 h, RT). Protein bands were visualized with ECL reagent (NCM Biotech, Suzhou, China) and quantified using ImageJ (version 1.52a, NIH, Bethesda, MD, USA). Primary antibodies used were: IL-17RA (1:1000, DF3602, Affinity, Nanjing, China), ACT1 (1:1000, ab137395, Abcam, London, UK), TRAF6 (1:1000, AF5376, Affinity, Nanjing, China), IκBα (1:1000, AF5002, Affinity, Nanjing, China), p-IκBα (1:1000, AF2002, Affinity, Nanjing, China), p65 (1:5000, 10745-1-AP, Proteintech, Wuhan, China), p-p65 (1:5000, 82335-1-RR, Proteintech, Wuhan, China), COX2 (1:2000, sc-19999, Santa Cruz, TX, USA), INOS (1:2000, sc-7271, Santa Cruz, TX, USA), ERK1/2 (1:2000, 4695T, CST, Danvers, MA, USA), p-ERK1/2 (1:2000, 4370T, CST, Danvers, MA, USA), JNK (1:2000, 9252T, CST Danvers, MA, USA), p38 (1:2000, 8690T, CST, Danvers, MA, USA), p-JNK (1:2000, 80024-1-RR, Proteintech, Wuhan, China), p-p38 (1:2000, 28796-1-AP, Proteintech, Wuhan, China).

### 2.8. RNA Sequencing and Analysis

Total RNA was extracted from BMECs using the TRIzol method. After quality assessment, library construction and sequencing were performed by Shanghai Applied Protein Technology Co., Ltd. (Shanghai, China). The library construction process included: mRNA enrichment using Oligo magnetic beads, fragmentation, reverse transcription into double-stranded cDNA, end repair, A-tailing, adapter ligation, and PCR amplification. The libraries were sequenced on an Illumina platform (Illumina, San Diego, CA, USA) with paired-end reads. Raw reads were filtered to remove adapter sequences and low-quality reads, then aligned to the bovine reference genome (Bos taurus Ensembl 104) using HISAT2. Gene quantification was performed with feature. Counts and normalized to FPKM. Differentially expressed genes were identified using DESeq2 with thresholds of |log_2_FC| > 1 and padj < 0.05. GO and KEGG enrichment analyses were performed using Fisher’s exact test with a significance threshold of *p* < 0.05.

### 2.9. Histopathological Analysis

To examine pathological alterations, both colon tissues and organoids were fixed in 4% formalin, samples were dehydrated, embedded in paraffin, and cut into 5 μm sections. Sections were then deparaffinized in xylene, rehydrated using a graded alcohol series, and subsequently stained with hematoxylin and eosin (H&E). A masked histopathological assessment was then conducted under a light microscope.

### 2.10. Histological Scoring of Mammary Tissue

Mammary tissue sections were stained with H&E and evaluated by a pathologist blinded to group allocation. Histological scoring was performed according to the method described by Zhao et al. [28]. Briefly, two parameters were each scored on a scale of 0–3:

Destruction of acinar structure: 0 = no signs of destruction; 1 = slight destruction; 2 = moderate destruction; 3 = severe structure destruction.

Inflammatory cell infiltration: 0 = no cell infiltration; 1 = slight; 2 = moderate; 3 = severe.

The two scores were summed to obtain a total histological score (0–6) for each animal.

### 2.11. Immunohistochemistry

Deparaffinized and rehydrated sections underwent antigen retrieval using citrate buffer (pH 6.0) by microwave heating. Following blockade with 3% goat serum, sections were incubated at 4 °C overnight with primary antibodies: anti-IL-17RA (1:200, DF3602, Affinity, Nanjing, China), anti-ACT1 (1:200, ab137395, Abcam, Cambridge, UK), and anti-TRAF6 (1:200, AF5376, Affinity, Nanjing, China), then exposed to HRP-conjugated secondary antibody for 2 h at RT. Signals were developed using a DAB kit (CST, Danvers, MA, USA), and sections were counterstained with hematoxylin. After dehydration and clearing, sections were examined under a light microscope (Olympus, Tokyo, Japan).

### 2.12. Immunofluorescent Staining

For immunofluorescence staining of NF-κB p65, BMECs cultured on glass coverslips were fixed using 4% paraformaldehyde. Following fixation, the cells were permeabilized with 0.1% Triton X-100 and blocked with 10% FBS in PBS for 1 h at room temperature. Next, the cells were incubated overnight at 4 °C with an anti-NF-κB p65 primary antibody (1:200, 82335-1-RR, Proteintech, Wuhan, China). Following five washes with PBS, the cells were incubated with Alexa Fluor 594-conjugated secondary antibody (A-11012, Thermo Fisher Scientific, Waltham, MA, USA) over a period of 1–2 h at RT, counterstained with DAPI, and examined for NF-κB p65 subcellular localization under a fluorescence microscope.

### 2.13. Statistical Analysis

All data are expressed as mean ± SEM. All experiments were performed with biological replicates, and the sample size (*n*) represents the number of independent biological replicates. For in vivo mouse experiments, *n* refers to the number of mice per group. For in vitro cell experiments, *n* refers to the number of independent experimental repetitions. Detailed sample size for each experiment is indicated in the corresponding figure legends. Statistical analyses were conducted using GraphPad Prism 9 (GraphPad Software, San Diego, CA, USA) via one-way ANOVA followed by Dunnett’s post hoc test for comparisons to the control group. Exact *p* values are indicated in the figures; for *p* values less than or equal to 0.0001, they are uniformly denoted as “*p* = 0.0001” on the graphs. A two-tailed *p* < 0.05 was considered statistically significant, and *p* < 0.01 was considered highly significant.

## 3. Results

### 3.1. PSB4 Inhibited the LPS-Stimulated BMECs’ Inflammatory Reaction

**Figure 1:**
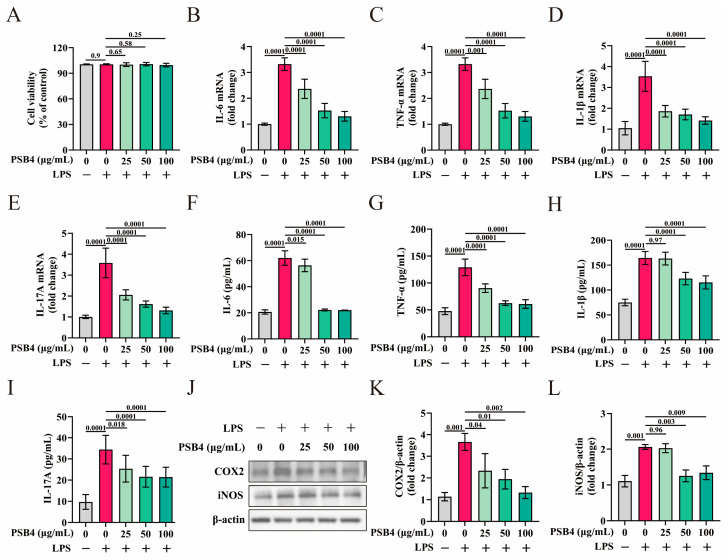
PSB4 inhibited inflammatory factors and mediators expression in LPS-induced BMECs. BMECs were seeded into culture plates and allowed to adhere, the cells were then exposed to LPS (10 μg/mL) for 6 h, followed by treatment with different concentrations of PSB4 (25, 50, and 100 μg/mL) for an additional 18 h without changing the culture medium. (**A**) CCK-8 method was employed to assess the cytotoxicity of varying concentrations of PSB4 on BMECs. (**B**–**E**) The impact of PSB4 on mRNA levels of *IL-6* (**B**), *TNF-α* (**C**), *IL-1β* (**D**) and *IL-17A* (**E**) was detected by qRT-PCR (*n* = 3). (**F**–**I**) The concentrations of IL-6 (**F**), TNF-α (**G**), IL-1β (**H**), and IL-17A (**I**) in the culture supernatants from LPS-induced BMECs were determined by ELISA (*n* = 3). (**J**–**L**) Expression of COX2 and iNOS proteins was analyzed by Western blotting (*n* = 3). Results are represented as mean ± SEM. *p* < 0.05 or *p* < 0.01 indicates that the difference between the labeled groups is significant or highly significant. Exact *p* values are indicated in the figures; for *p* values less than or equal to 0.0001, they are uniformly denoted as “*p* = 0.0001” on the graphs.

To evaluate the anti-inflammatory effects of PSB4, we established an inflammatory model using LPS-induced BMECs. As depicted in Figure 1A, the cytotoxicity test showed that the selected concentrations of PSB4 had no significant effect on BMEC viability, either in the presence or absence of LPS treatment (*p* > 0.05). Compared with the control group, LPS dramatically up-regulated mRNA levels of *IL-6*, *TNF-α*, *IL-1β*, and *IL-17A* (*p* < 0.01), while PSB4 significantly inhibited cytokine expression dose-dependently (Figure 1B–E, *p* < 0.01). At the same time, the results of ELISA also exhibited a pattern consistent with the transcription level (Figure 1F–I, *p* < 0.01). Furthermore, PSB4 markedly suppressed the levels of inflammatory mediators (COX2 and iNOS) under LPS stimulation showing dose dependency (Figure 1J–L, *p* < 0.01).

### 3.2. PSB4 Inhibits IL-17RA Signaling and Its Downstream MAPK/NF-κB Pathways in Murine Mastitis Models

**Figure 2:**
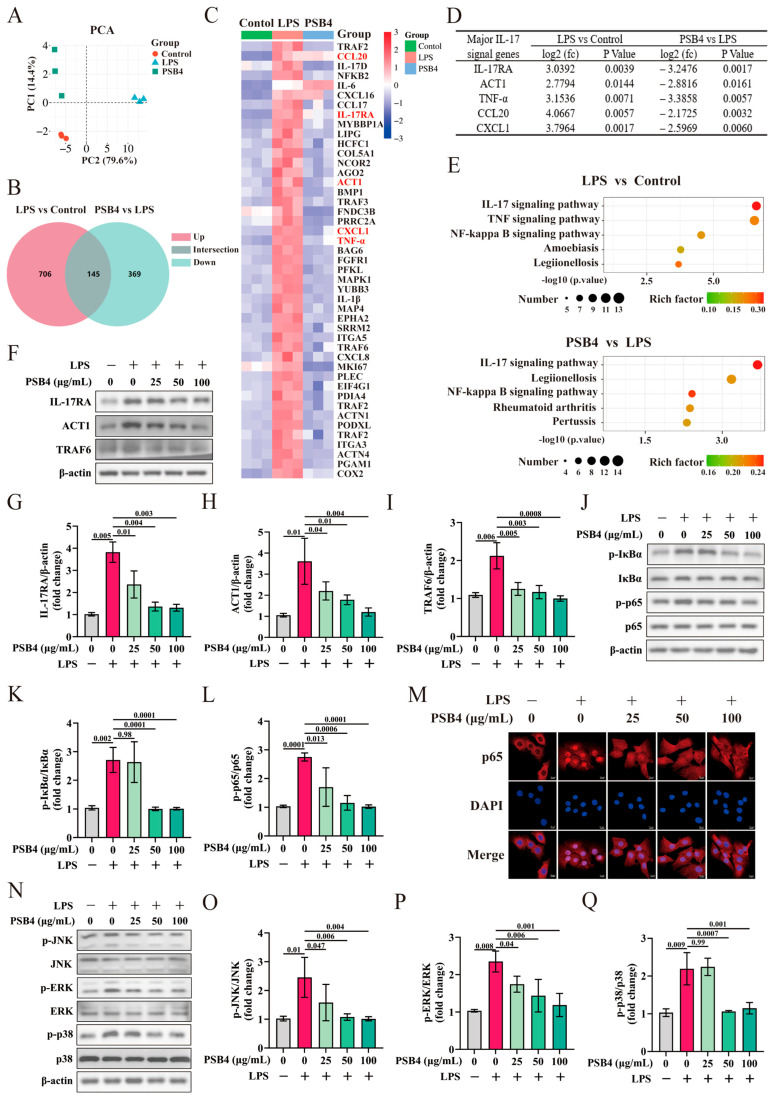
The regulatory impact of PSB4 on the IL-17RA axis and its downstream cascades in LPS-treated BMECs. BMECs were seeded into culture plates and allowed to adhere, the cells were then exposed to LPS (10 μg/mL) for 6 h, followed by treatment with different concentrations of PSB4 (25, 50, and 100 μg/mL) for an additional 18 h without changing the culture medium. (**A**) Two-dimensional principal component analysis (PCA) of control, LPS-treated, and LPS + PSB4-treated samples. (**B**) Venn diagram of differential genes in different comparison groups. (**C**) Heatmap depicting IL-17RA signaling and its downstream cascades related-genes (Red indicates significantly different genes in the IL-17RA pathway among treatment groups.). (**D**) The fold change (fc), *p* value and false discovery rate (FDR) of IL-17RA signaling key genes. (**E**) Differential abundance score of enriched KEGG pathways. (**F**–**I**) Protein levels of IL-17RA signaling core components (IL-17RA, ACT1 and TRAF6) in the BMECs were assessed by immunoblotting (*n* = 3). (**J**–**L**) The abundance of NF-κB pathway proteins (p-IκBα and p-p65) in the BMECs was evaluated by Western blotting (*n* = 3). (**M**) Immunofluorescence analysis of NF-κBp65 (red) and DAPI (blue) nuclear staining (Bar = 10 μm). (**N**–**Q**) The levels of MAPK cascade components (p-JNK, p-ERK and p-p38) in the BMECs were determined using Western blotting (*n* = 3). All data are presented as mean ± SEM. *p* < 0.05 or *p* < 0.01 indicates that the difference between the labeled groups is significant or highly significant. Exact *p* values are indicated in the figures; for *p* values less than or equal to 0.0001, they are uniformly denoted as “*p* = 0.0001” on the graphs.

To elucidate how PSB4 suppresses LPS-induced inflammatory responses in BMECs, RNA-seq analysis was performed on BMECs among the Control group, LPS group, and LPS + PSB4 (100 μg/mL) group. As shown in Figure 2A–E, interrogation of the Venn diagram suggests that PSB4 treatment reverses a significant number of LPS-induced gene expression alterations. KEGG analysis implicated IL-17RA, TNF, and NF-κB signaling in the response to LPS stimulation. Interestingly, PSB4 treatment interfered with the activity of the IL-17RA signaling and the NF-κB pathway (Figure 2E, *p* < 0.01). To further clarify the signaling pathways through which PSB4 exerts its effects, we evaluated the differential gene expression between the groups. LPS stimulation markedly upregulated IL-17RA pathway-associated genes (*IL-17RA*, *ACT1*, *TNF-α*, *CXCL1* and *CCL20*) in BMECs, while PSB4 treatment reversed this effect (Figure 2C,D, *p* < 0.01). This indicates that PSB4 exerts anti-inflammatory effects may be associated with IL-17RA signaling. To explore the impact of PSB4 on the IL-17RA axis and downstream signaling. The protein abundance of IL-17RA, ACT1, TRAF6, p-ERK, p-JNK, p-p38, p-p65 and p-I*κ*B*α* in BMECs was quantified. As depicted in Figure 2F–L and Figure 2N–Q, LPS activated the IL-17RA signaling (IL-17RA, ACT1, TRAF6), the MAPK cascade (p-ERK, p-JNK, p-p38) and the NF-κB pathway (p-p65, p-IκBα) (*p* < 0.01), while PSB4 inhibited the activation of these signals in a dose-dependent manner (*p* < 0.01). Furthermore, immunofluorescence labeling revealed that PSB4 significantly inhibited LPS-induced nuclear accumulation of p65 (Figure 2M).

### 3.3. PSB4 Ameliorates rIL-17A-Induced Inflammation in BMECs

**Figure 3:**
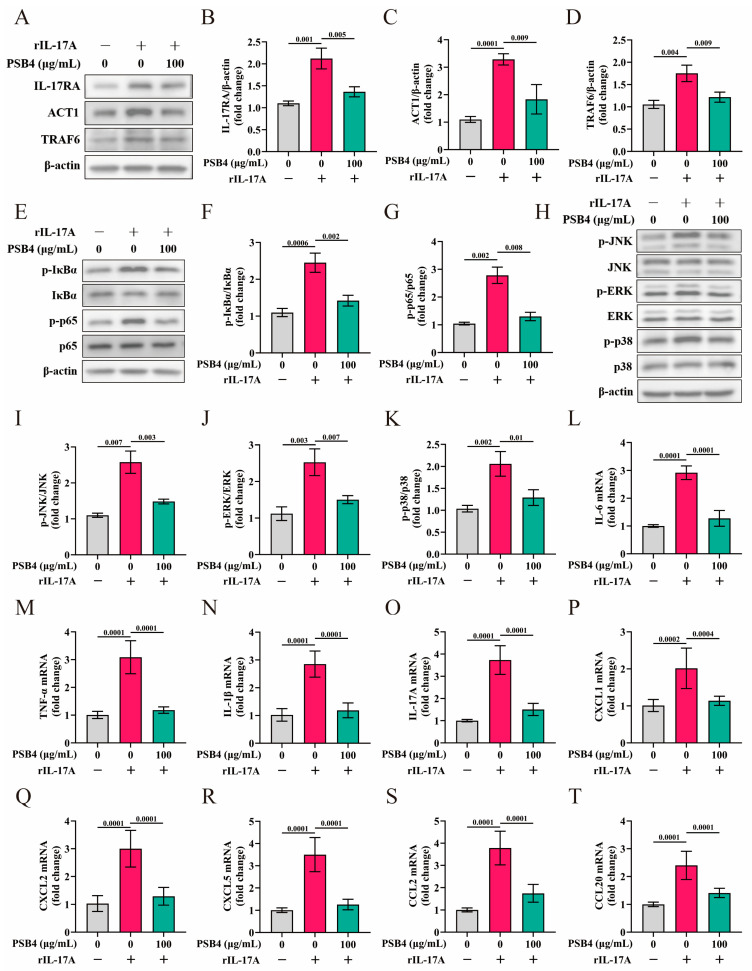
PSB4 suppressed the rIL-17A-induced activation of the IL-17RA axis and its downstream cascades in BMEC cultures. BMECs were seeded into culture plates and allowed to adhere, the cells were then exposed to rIL-17A (1 μg/mL) for 6 h, followed by treatment with PSB4 (100 μg/mL) for an additional 18 h without changing the culture medium. (**A**–**D**) Protein abundance of IL-17RA signaling core components (IL-17RA, ACT1 and TRAF6) in the BMECs was assessed by immunoblotting (*n* = 3). (**E**–**G**) Levels of NF-κB pathway proteins (p-IκBα and p-p65) in the BMECs were evaluated via Western blotting (*n* = 3). (**H**–**K**) The expression levels of MAPK cascade members (p-JNK, p-ERK and p-p38) in the BMECs were quantified using Western blot analysis (*n* = 3). (**L**–**O**) qRT-PCR was employed to assess the mRNA levels of *IL-6* (**L**), *TNF-α* (**M**), *IL-1β* (**N**) and *IL-17A* (**O**), which are downstream inflammatory factors of the IL-17RA axis (*n* = 3). (**P**–**T**) The same method was used to quantify the mRNA expression of chemokines *CXCL1* (**P**), *CXCL2* (**Q**), *CXCL5* (**R**), *CCL2* (**S**) and *CCL20* (**T**) downstream of IL-17RA signaling (*n* = 3). All values are expressed as mean ± SEM. *p* < 0.05 or *p* < 0.01 indicates that the difference between the labeled groups is significant or highly significant. Exact *p* values are indicated in the figures; for *p* values less than or equal to 0.0001, they are uniformly denoted as “*p* = 0.0001” on the graphs.

Given the potential of PSB4 to alleviate LPS-elicited inflammatory reaction in BMECs through suppression of the IL-17RA and NF-κB pathways, we established an rIL-17A-induced inflammatory model to complementarily investigate its modulatory effects. As expected, PSB4 significantly inhibited the rIL-17A-induced elevation of key proteins in the IL-17RA signaling (IL-17RA, ACT1, TRAF6), the MAPK cascade (p-ERK, p-JNK, p-p38) and the NF-κB pathway (p-p65, p-IκBα) as well as the synthesis of downstream pro-inflammatory factors (*IL-6*, *TNF-α*, *IL-1β*, and *IL-17A*) and chemokines (*CXCL1*, *CXCL2*, *CXCL5*, *CCL2* and *CCL20*) (Figure 3A–T, *p* < 0.01).

### 3.4. PSB4 Mitigates LPS-Induced Inflammation in BMECs Through Suppression of the IL-17RA Axis and Downstream MAPK/NF-κB Pathway

**Figure 4:**
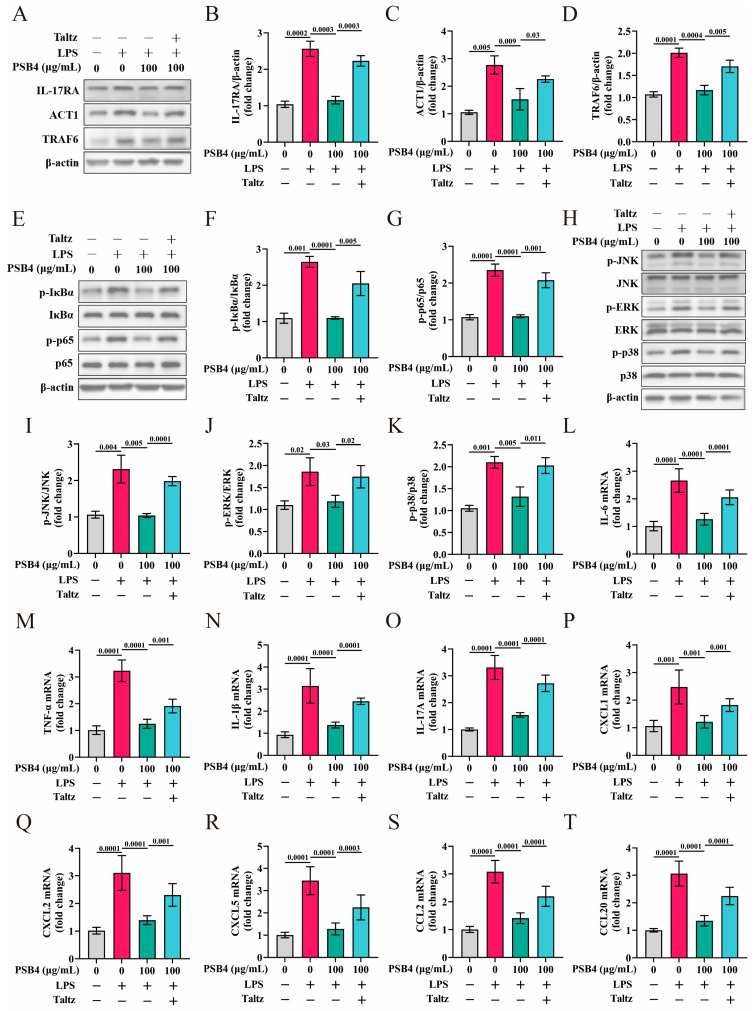
PSB4 alleviates LPS-elicited inflammation in BMECs by inhibiting IL-17RA and its downstream signaling. BMECs were seeded into culture plates and allowed to adhere, the cells were then exposed to LPS (10 μg/mL) for 4 h, followed by exposure to Taltz (10 μg/mL) for 2 h, and then further incubated with PSB4 (100 μg/mL) for an additional 18 h. The culture medium was not changed or replenished during the entire treatment period. (**A**–**D**) Protein levels of IL-17RA pathway core components (IL-17RA, ACT1 and TRAF6) in the BMECs were assessed via Western blot analysis. (**E**–**G**) Protein abundance of NF-κB pathway core components (p-IκBα and p-p65) in the BMECs was assessed via Western blot analysis (*n* = 3). (**H**–**K**) Levels of MAPK cascade key proteins (p-ERK, p-JNK and p-p38) within the BMECs were evaluated using Western blot analysis (*n* = 3). (**L**–**O**) qRT-PCR was employed to measure the transcript levels of *IL-6* (**L**), *TNF-α* (**M**), *IL-1β* (**N**) and *IL-17A* (**O**), which are downstream inflammatory mediators of IL-17RA signaling (*n* = 3). (**P**–**T**) qRT-PCR was employed to measure the transcript levels of chemokines *CXCL1* (**P**), *CXCL2* (**Q**), *CXCL5* (**R**), *CCL2* (**S**) and *CCL20* (**T**) downstream of IL-17RA signaling (*n* = 3). All values are expressed as mean ± SEM. *p* < 0.05 or *p* < 0.01 indicates that the difference between the labeled groups is significant or highly significant. Exact *p* values are indicated in the figures; for *p* values less than or equal to 0.0001, they are uniformly denoted as “*p* = 0.0001” on the graphs.

To further demonstrate that PSB4 alleviates LPS-induced inflammation in BMECs via IL-17RA signaling, BMECs were co-treated with Taltz (Ixekizumab, an IgG4 monoclonal antibody that selectively blocks the interaction between IL-17A and its cognate receptor IL-17RA) and PSB4. As depicted in Figure 4A–K, PSB4 significantly counteracted the LPS-induced upregulation of key proteins in the IL-17RA (IL-17RA, TRAF6 and ACT1), NF-κB (p-p65 and p-IκBα), and MAPK (p-ERK, p-JNK and p-p38) pathways (*p* < 0.01). However, co-administration of Taltz with PSB4 effectively reversed this inhibitory action (*p* < 0.01). Furthermore, as illustrated in Figure 4L–T, the transcript levels of downstream inflammatory cytokines (*IL-6*, *TNF-α*, *IL-1β*, and *IL-17A*) and chemotactic factors (*CXCL1*, *CXCL2*, *CXCL5*, *CCL2* and *CCL20*) in the IL-17RA pathway exhibited a trend consistent with the changes in key pathway proteins.

### 3.5. PSB4 Alleviated LPS-Induced Inflammation of Mammary Gland in Mice

**Figure 5:**
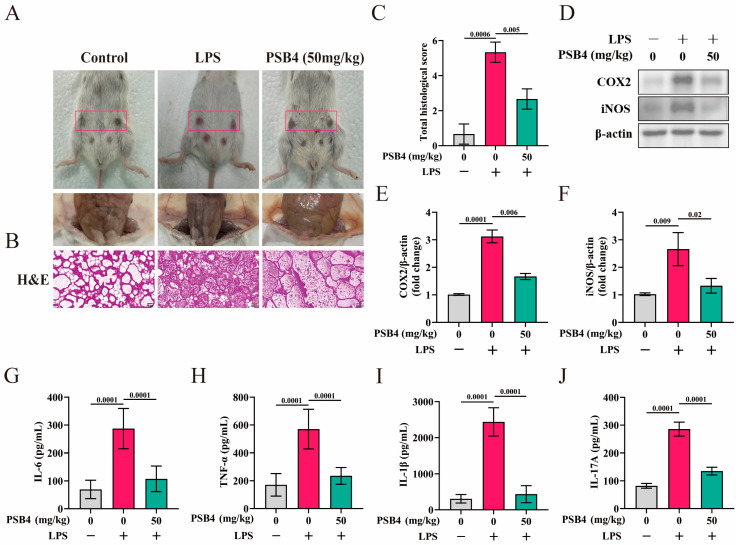
PSB4 suppresses the upregulation of pro-inflammatory factors in serum and inflammatory mediator proteins in mammary tissue of LPS-challenged mice. On lactation days 3, 5, and 7, both LPS-treated and LPS + PSB4-treated mice underwent intramammary administration of LPS (0.2 mg/mL, 50 μL) into the fourth mammary glands. Starting from lactation day 3, the LPS + PSB4 group received PSB4 (50 mg/kg/day) once daily via intraperitoneal injection for 7 consecutive days. Control animals were given an equivalent volume of saline via intraperitoneal injection once daily from day 3, also for 7 consecutive days. (**A**) Gross pathological images of the fourth mammary gland across treatment groups (The red box indicates the nipple through which LPS was injected to establish the mastitis model). (**B**) Histological evaluation of mammary tissue performed on H&E-stained sections (scale bar = 50 μm). (**C**) Total histopathological scores of representative mammary tissue samples (*n* = 3 randomly selected mice per group). (**D**–**F**) Immunoblotting analysis of COX2 and iNOS protein abundance in representative mammary tissue samples (*n* = 3 randomly selected mice per group). (**G**–**J**) ELISA was employed to assess the impact of PSB4 on the concentrations of IL-6 (**G**), TNF-α (**H**), IL-1β (**I**) and IL-17A (**J**) in serum of mastitis mice (*n* = 12). All values are expressed as mean ± SEM. *p* < 0.05 or *p* < 0.01 indicates that the difference between the labeled groups is significant or highly significant. Exact *p* values are indicated in the figures; for *p* values less than or equal to 0.0001, they are uniformly denoted as “*p* = 0.0001” on the graphs.

To assess the protective effects of PSB4 and its underlying mechanisms, we established LPS-induced mastitis mouse model. As shown in Figure 5A,B. Anatomical observations revealed that mammary glands in the control group exhibited typical semi-translucent, milky-white appearance, smooth surface, and uniform soft texture. In contrast, LPS-induced mice presented markedly swollen and opaque mammary glands with a dark red coloration, indicating severe hyperemia and inflammatory infiltration. These inflammatory manifestations were significantly alleviated by PSB4 treatment. H&E staining revealed well-preserved alveolar structures in the control group, whereas the LPS group displayed extensive inflammatory cell infiltration and destruction of mammary tissue architecture. PSB4 administration markedly ameliorated LPS-induced inflammatory cell accumulation and tissue damage. Semi-quantitative histopathological scoring confirmed these observations. As shown in Figure 5C, the total histopathological score was significantly higher in the LPS group (5.33 ± 0.33) than in the control group (0.67 ± 0.33, *p* < 0.01), and PSB4 treatment significantly reduced the score (2.67 ± 0.33) compared with the LPS group (*p* < 0.01). Consistent with these findings, ELISA results demonstrated that LPS stimulation significantly elevated serum concentrations of inflammatory cytokines, including IL-6, TNF-α, IL-1β and IL-17A (*p* < 0.01), all of which were substantially suppressed by PSB4 treatment (Figure 5G–J, *p* < 0.01). Further supporting these observations, Western blot analysis demonstrated that LPS-induced upregulation of key inflammatory mediators (COX-2 and iNOS) in mammary tissue was significantly attenuated in PSB4-treated mice (Figure 5D–F, *p* < 0.01).

### 3.6. PSB4 Inhibits the IL-17RA Axis and Its Downstream MAPK/NF-κB Pathways in Murine Mastitis Models

**Figure 6:**
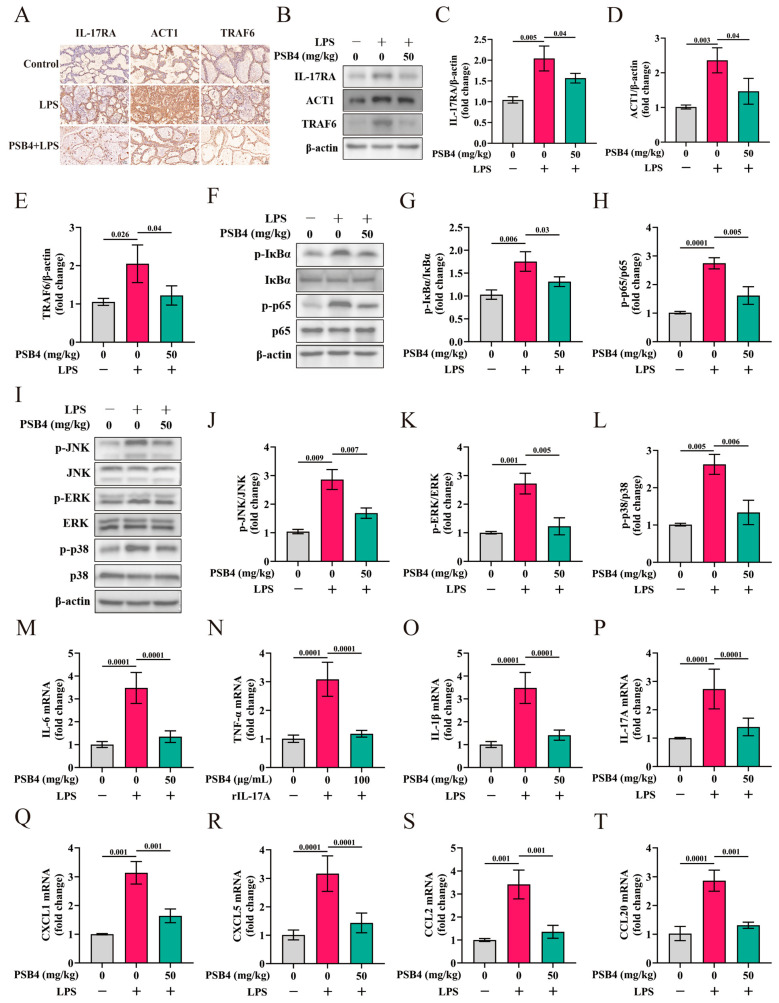
PSB4 alleviates LPS-elicited mastitis in murine models through suppression of the IL-17RA pathway and its downstream MAPK/NF-κB signaling. On lactation days 3, 5, and 7, mice in both the LPS-treated and LPS + PSB4-treated cohorts underwent intramammary administration of LPS (0.2 mg/mL, 50 μL) into the fourth mammary glands. Starting from lactation day 3, the LPS + PSB4 group was administered PSB4 (50 mg/kg/day) once daily via intraperitoneal injection for 7 consecutive days. The Control group received an equivalent volume of saline through intraperitoneal injection once daily from day 3, also for 7 consecutive days. (**A**) Immunohistochemical detection of IL-17RA, ACT1 and TRAF6 proteins in mouse breast tissue. (**B**–**E**) Representative Western blot analysis of IL-17RA pathway core components (IL-17RA, ACT1 and TRAF6) in mouse mammary tissues (*n* = 3 randomly selected mice per group). (**F**–**H**) Representative Western blot analysis of NF-κB signaling proteins (p-IκBα and p-p65) in mouse mammary tissues (*n* = 3 randomly selected mice per group). (**I**–**L**) Representative Western blot analysis of MAPK cascade mediators (p-ERK, p-JNK and p-p38) in mouse mammary tissues (*n* = 3 randomly selected mice per group). (**M**–**P**) qRT-PCR was employed to measure the transcript levels of *IL-6* (**M**), *TNF-α* (**N**), *IL-1β* (**O**) and *IL-17A* (**P**), which are downstream inflammatory mediators of the IL-17RA axis (*n* = 12). (**Q**–**T**) The same method was used to quantify the mRNA expression of chemokines *CXCL1* (**Q**), *CXCL5* (R), *CCL2* (**S**) and *CCL20* (**T**) downstream of IL-17RA signaling (*n* = 12). All values are expressed as mean ± SEM. *p* < 0.05 or *p* < 0.01 indicates that the difference between the labeled groups is significant or highly significant. Exact *p* values are indicated in the figures; for *p* values less than or equal to 0.0001, they are uniformly denoted as “*p* = 0.0001” on the graphs.

Building on previous in vitro findings that PSB4 inhibits IL-17RA and downstream MAPK/NF-κB signaling, we validated this mechanism in a murine mastitis model. Immunohistochemistry showed that LPS challenge strongly enhanced positive signals for IL-17RA, ACT1, and TRAF6 in mammary tissue, whereas PSB4 treatment markedly suppressed their expression (Figure 6A). Immunoblotting analysis further demonstrated that LPS significantly upregulated the abundance of IL-17RA pathway mediators (IL-17RA, ACT1, TRAF6) as well as downstream MAPK (p-ERK, p-JNK, p-p38) and NF-κB (p-p65, p-IκBα) signaling proteins (*p* < 0.01). PSB4 administration significantly counteracted these changes (*p* < 0.01), demonstrating its ability to attenuate IL-17RA-driven signaling in vivo (Figure 6B–L). In addition, qRT-PCR revealed that LPS-induced induction of inflammatory cytokines (*IL-6*, *TNF-α*, *IL-1β* and *IL-17A*), as well as chemotactic factors (*CXCL1*, *CXCL5*, *CCL2* and *CCL20*), was significantly suppressed by PSB4 (*p* < 0.01), indicating transcriptional downregulation of these inflammatory mediators (Figure 6M–T).

## 4. Discussion

Bovine mastitis continues to be a highly common and financially damaging illness impacting the worldwide dairy sector. The conventional approach to managing bovine mastitis predominantly depends on antimicrobials. Nevertheless, the inappropriate application of these drugs leads to their accumulation in milk and dairy, posing food safety risks, while also contributing to bacterial resistance and complicating mastitis control. Hence, identifying safe and potent antibiotic substitutes is a critical industry priority. In recent years, natural products, particularly bioactive plant-derived compounds, have been widely investigated for mastitis treatment [29,30]. In this research, we evaluated the effect of PSB4 on mammary gland inflammation and its underlying mechanisms in LPS-induced mammary epithelial cells and a murine mastitis model. The results indicate that PSB4 significantly inhibits the inflammatory response, and this effect is achieved by the suppression of IL-17RA signaling and its downstream MAPK/NF-κB pathways.

BMECs serve not only as functional units within mammary tissue, but also as key participants and coordinators in the bovine mammary defense system [31,32]. *Escherichia coli*, a Gram-negative bacterium, ranks among the leading causes of bovine mastitis [33,34]. LPS, a potent virulence factor produced by Gram-negative bacterium, is capable of eliciting a severe inflammatory response within mammary tissues [35,36]. The central pathogenic mechanism involves the release of LPS, which activates the innate immune recognition pathways in mammary tissue, triggering an inflammatory cascade characterized by cytokine storm and immune cell infiltration. This ultimately culminates in a localized inflammatory response and mastitis. The inflammatory process follows a defined sequence: it begins with the release of pathogen virulence factors, which triggers immune recognition. This recognition activates intracellular signal transduction pathways, leading to a robust amplification of the inflammatory cascade, and ultimately culminates in tissue damage. Key inflammatory mediators, including TNF-α, IL-1β, and IL-6, function as critical effector molecules within this cascade and are regulated by its central signaling hub. Consequently, targeted blockade of these inflammatory cytokines represents a viable strategy for mitigating inflammatory responses and alleviating disease progression [37,38]. Our results demonstrated that PSB4 significantly reduced the expression of inflammatory factors (IL-6, TNF-α, IL-1β and IL-17A) and mediators (iNOS and COX-2) in LPS-induced BMECs. These results indicate that PSB4 may serve as a promising candidate for mastitis treatment This finding aligns with the recent clinical study by Zhang et al. (2025), in which PSB4 treatment (via intramuscular injection for 7 days) in cows with clinical mastitis significantly reduced serum levels of IL-1β, IL-6, and TNF-α, restoring these cytokines to levels comparable to those in healthy controls [39].

Although previous studies have demonstrated that PSB4 modulates inflammatory pathways to suppress the levels of pro-inflammatory factors and mediators, thereby treating inflammation-related diseases, its specific molecular mechanism in inflammation of mammary gland remains unclear [19,20]. To investigate the protective mechanism of PSB4 in counteracting LPS-triggered inflammation within BMECs, we per-formed transcriptomic analysis. The results revealed that its action is associated with the IL-17RA signaling. It is well-established that IL-17 signaling does not function as an independent, strong signaling pathway but rather acts as a subtle “signal amplifier.” It regulates downstream MAPK and NF-κB signaling, inducing the production of various inflammatory mediators and chemotactic factors, thereby amplifying the immune cascade. Precisely, LPS stimulation activates the natural immune system, triggering a massive production of inflammatory molecules, thereby inducing the upregulation of the IL-17RA signaling. This signal subsequently engages the ACT1/TRAF6 axis to con-currently initiate the downstream MAPK and NF-κB pathways. While the swift induction of the NF-κB pathway potently drives the initial transcription of inflammation-related genes, the MAPK pathway amplifies and stabilizes their expression through enhanced post-transcriptional regulation. This synergistic action promotes an explosive increase in the synthesis of inflammatory factors and chemokines. Notably, certain newly produced factors, such as TNF-α, can in turn feedback to reactivate the IL-17RA signaling, forming a self-amplifying positive feedback loop. It is this dual mechanism that drives the IL-17RA signal to drive a strong and persistent inflammatory response [40,41]. Therefore, regulating IL-17RA signaling is a key target for alleviating mammary gland inflammation. Our results showed that PSB4 treatment inhibited the LPS-induced activation of the IL-17RA axis and the downstream MAPK/NF-κB pathway-related proteins in BMECs. To further determine whether the inflammation-dampening effect of PSB4 depends on the IL-17RA pathway, we first induced inflammation in BMECs using rIL-17A. Our results indicated that PSB4 markedly suppressed the rIL-17A-induced upregulation of key proteins in the IL-17RA signaling cascade and the subsequent MAPK/NF-κB pathways, as well as the expression of downstream factors. This finding directly demonstrates that PSB4 can counteract IL-17RA-mediated signaling activation. Similar inhibitory effects were consistently observed in the expression of downstream pro-inflammatory factors and chemokines. More importantly, we then disrupted the IL-17A/IL-17RA axis using the IL-17A inhibitor Taltz. Under this condition, the ability of PSB4 to inhibit the LPS-induced upregulation of IL-17RA, MAPK, and NF-κB pathway proteins, as well as their downstream inflammatory cytokines and chemokines, was almost completely reversed. Collectively, these results confirm that PSB4 achieves its anti-inflammatory effects by specifically suppressing the IL-17RA signaling pathway and its downstream MAPK/NF-κB cascades.

In another study, we demonstrated that PSB4 injection effectively alleviated clinical signs in cows with clinical mastitis. This treatment simultaneously reduced pro-inflammatory cytokine levels, milk somatic cell score, as well as the prevalence of bacterial pathogens in milk [39]. The therapeutic mechanism of PSB4, however, warrants further investigation. Serving as a critical bridge from mechanistic discovery to clinical application in bovine mastitis treatment, the mouse mastitis model efficiently and reproducibly simulates the core inflammatory pathology of the mammary gland, including immune cell infiltration, a pro-inflammatory cytokine storm, and tissue damage, under genetically uniform and highly controlled conditions [42,43,44]. This controlled system enables the precise dissection of PSB4’s mechanism of action in alleviating mastitis, effectively isolating its effects from confounding variables such as individual variation, lactation stage, and herd management practices. Hence, we employed an LPS-induced mouse mastitis model to evaluate the in vivo effects of PSB4. In this model, PSB4 treatment reduced inflammatory cytokine levels (IL-17A, IL-6, IL-1β and TNF-α), decreased inflammatory mediator protein expression (COX2 and iNOS), and attenuated mammary tissue pathological changes. Furthermore, we found that PSB4 reduced the protein levels of IL-17RA, ACT1 and TRAF6, as well as the phosphorylation of MAPK and NF-κB pathway components, and suppressed the mRNA expression of downstream inflammatory cytokines and chemokines in this model. These in vivo findings closely mirror our in vitro results, confirming that the protective effect of PSB4 against mammary inflammatory injury is mediated through the inhibition of the IL-17RA signaling pathway and its downstream MAPK/NF-κB cascades. Notably, other triterpenoid saponins have been similarly evaluated using the mouse mastitis model, further supporting the reliability of this experimental system. For instance, Saiko saponin A, a triterpenoid saponin isolated from Bupleurum falcatum, has been demonstrated to effectively alleviate Staphylococcus aureus-induced mastitis in mice by inhibiting NF-κB pathway activation and attenuating the inflammatory response, with significantly reduced TNF-α and IL-1β levels observed in the mammary tissue of treated groups [45]. These independent findings on structurally related saponins collectively corroborate the anti-inflammatory potential of this compound class in mammary tissue. These results demonstrate that PSB4 can effectively suppress mammary inflammation and tissue damage, positioning it as a promising therapeutic agent against bovine mastitis. This finding is consistent with previous studies [46,47].

However, we acknowledge that caution is warranted when translating these findings from a mouse model to dairy cows, given inherent species differences and model limitations. Mouse and bovine mammary glands differ markedly in anatomical structure, immune cell composition, and drug metabolism characteristics; consequently, the effective dose determined in mice may not directly predict the optimal therapeutic dose in cattle without dedicated target-animal pharmacokinetic/pharmacodynamic bridging studies. The LPS-induced mouse mastitis model used in this study has been widely employed as a standardized and reproducible system for investigating mammary inflammatory signaling mechanisms and for the preclinical evaluation of candidate therapeutics for bovine mastitis, because the core NF-κB-driven inflammatory cascade is conserved across mammalian species [48]. Moreover, directly conducting experimental studies in dairy cows is often constrained by high costs, operational difficulties, and challenges in standardization, whereas mouse models offer advantages of low cost, short experimental duration, high throughput, and good reproducibility, enabling the systematic screening of pharmacological interventions under well-controlled conditions [49]. LPS, as the key pathogen-associated molecular pattern of Gram-negative bacteria, is a primary trigger of the inflammatory responses observed in clinical coliform mastitis; therefore, LPS infusion into the mammary gland provides a well-defined, acute inflammatory model that specifically isolates the endotoxin-driven inflammatory component from the complex variables of live bacterial infection, including differential pathogen virulence, inoculum variability, and host–pathogen interactions. This model system has been demonstrated to recapitulate pathological features similar to the lesions observed in mastitis of dairy cattle. Our findings clarify the specific anti-inflammatory mechanisms of PSB4 in the mammary gland by demonstrating its inhibition of the NF-κB signaling pathway, identify key molecular targets (e.g., TLR4, NF-κB, and downstream pro-inflammatory cytokines) for therapeutic intervention, and provide a pharmacological rationale for developing PSB4 as an adjunctive anti-inflammatory agent in mastitis management. These data also establish the effective dose range and treatment window in vivo, which will inform the design of subsequent target-animal studies. Nonetheless, we fully recognize that the LPS model cannot directly evaluate the bactericidal or bacteriostatic function of PSB4, nor can it fully recapitulate the entire spectrum of naturally occurring mastitis, which often involves polymicrobial infections characterized by complex host–pathogen interactions, biofilm formation, and chronic disease progression. Alleviating inflammation without eliminating the infectious etiology may lead to persistent subclinical mastitis. Future studies employing live bacterial challenge models in cattle, followed by well-designed field trials, will be required to validate the clinical applicability of these mechanistic findings and to determine whether PSB4 should be used as monotherapy or in combination with antimicrobial agents.

## 5. Conclusions

Collectively, these findings demonstrate that PSB4 significantly alleviates LPS-triggered mastitis in experimental settings, encompassing both live subjects and cultured cells. Through combined in vitro and in vivo mechanistic verification, we reveal that the inflammation-dampening action of PSB4 is achieved via suppression of the IL-17RA signaling and its downstream MAPK/NF-κB cascades. This mechanistic insight not only provides a precise pharmacological basis for PSB4 in treating mammary inflammation, but also offers a strong theoretical framework for the design of novel anti-mastitis drugs targeting the IL-17RA pathway.

## Figures and Tables

**Table 1:** Primer sequences for bovine genes used in qPCR analysis.

Gene Name	Primer Sequences (5′ to 3′)	Product Length (bp)
*IL-1β*	F: AACGTCCTCCGACGAGTTTC R: CATGCAGAACACCACTTCTCG	160
*IL-6*	F: ACGAAAGAGAGCTCCATCTGC R: AATGGAGTGAAGGCGCTTGT	71
*IL-8*	F: CGAGGTCTGCCTAAACCCC R: TCTTGCTTCTCAGCTCTCTTCAC	78
*TNF-α*	F: CCCACGTTGTAGCCGACAT R: ATGAGGTAAAGCCCGTCAGC	133
*IL-17A*	F: CTTGGACTCTCCACCGCAATG R: TGATGGTCCACCTTCCCTTC	109
*CXCL1*	F: GACCAAACCGAAGTCATAGCC R: ACTTCACTTCCACTGAGGCTG	173
*CXCL2*	F: ACCAAACCGAAGTCATAGCCA R: CTGTAGGGGCAGGGTCTACT	175
*CXCL5*	F: TCCAAGGTGGAAGTGATAGCC R: TCAAAGGAGCTTCTGGGTCC	70
*CCL2*	F: AACATGAAGGTCTCCGCTGC R: TTGGGAGTTAATTGCATCTGGC	96
*CCL20*	F: GGCGAATCAGAAGCAAGCAA R: GGATTTGCGCACACAGACAA	164
*β-actin*	F: CACAGGCCTCTCGCCTTC R: ATCATCCATGGCGAACTGGT	71

**Table 2:** Primer sequences for mouse genes used in qPCR analysis.

Gene Name	Primer Sequences (5′ to 3′)	Product Length (bp)
*IL-6*	F: CCGGAGAGGAGACTTCACAG R: CAGAATTGCCATTGCACAAC	134
*IL-1β*	F: GCTGCTTCCAAACCTTTGAC R: AGCTTCTCCACAGCCCACAT	121
*TNF-α*	F: ACGGCATGGATCTCAAAGAC R: GTGGGTGAGGAGCACGTAGT	116
*IL-17A*	F: TCTCCACCGCAATGAAGAGT R: AAAGTGAAGGGGCAGCTCTC	174
*CXCL1*	F: CATGGCTGGGATTCACCTCA R: CCTCGCGACCATTCTTGAGT	107
*CXCL2*	F: CCTGCCAAGGGTTGACTTCA R: GCAAACTTTTTGACCGCCCT	112
*CXCL5*	F: GCTGGCATTTCTGTTGCTGT R: AAGCAAACACAACGCAGCTC	96
*CCL2*	F: CAGCCAGATGCAGTTAACGC R: GCTGCTGGTGATCCTCTTGC	102
*CCL20*	F: GTACTGCTGGCTCACCTCTG R: TCACAAGCTTCATCGGCCAT	131
*β-actin*	F: GTCAGGTCATCACTATCGGCAAT R: AGAGGTCTTTACGGATGTCAACGT	147

## Data Availability

The original contributions presented in this study are included in the article/Supplementary Materials. Further inquiries can be directed to the corresponding authors.

## Supplementary Materials

The following supporting information can be downloaded at: https://www.mdpi.com/article/10.3390/vetsci13060521/s1.

## References

[1] El-Sayed A., Kamel M. (2021). Bovine Mastitis Prevention and Control in the Post-Antibiotic Era. Trop. Anim. Health Prod..

[2] Ruegg P.L. (2017). A 100-Year Review: Mastitis Detection, Management, and Prevention. J. Dairy Sci..

[3] Ashraf A., Imran M. (2020). Causes, Types, Etiological Agents, Prevalence, Diagnosis, Treatment, Prevention, Effects on Human Health and Future Aspects of Bovine Mastitis. Anim. Health Res. Rev..

[4] Zalewska M., Brzozowska P., Rzewuska M., Kawecka-Grochocka E., Urbańska D.M., Sakowski T., Bagnicka E. (2025). The Quality and Technological Parameters of Milk Obtained from Dairy Cows with Subclinical Mastitis. J. Dairy Sci..

[5] Fadillah A., Van Den Borne B.H.P., Schukken Y.H., Poetri O.N., Hogeveen H. (2025). Cost-Efficiency of Mastitis Control Strategies on Smallholder Dairy Farms. J. Dairy Sci..

[6] Al-Farha A.A., Hemmatzadeh F., Khazandi M., Hoare A., Petrovski K. (2017). Evaluation of Effects of Mycoplasma Mastitis on Milk Composition in Dairy Cattle from South Australia. BMC Vet. Res..

[7] Puerto M.A., Shepley E., Cue R.I., Warner D., Dubuc J., Vasseur E. (2021). The Hidden Cost of Disease: I. Impact of the First Incidence of Mastitis on Production and Economic Indicators of Primiparous Dairy Cows. J. Dairy Sci..

[8] Sharun K., Dhama K., Tiwari R., Gugjoo M.B., Iqbal Yatoo M., Patel S.K., Pathak M., Karthik K., Khurana S.K., Singh R. (2021). Advances in Therapeutic and Managemental Approaches of Bovine Mastitis: A Comprehensive Review. Vet. Q..

[9] Saleem A., Saleem Bhat S., Omonijo F.A., Ganai N.A., Ibeagha-Awemu E.M., Mudasir Ahmad S. (2024). Immunotherapy in Mastitis: State of Knowledge, Research Gaps and Way Forward. Vet. Q..

[10] Berry S.P.D., Dossou C., Kashif A., Sharifinejad N., Azizi G., Hamedifar H., Sabzvari A., Zian Z. (2022). The Role of Il-17 and Anti-Il-17 Agents in the Immunopathogenesis and Management of Autoimmune and Inflammatory Diseases. Int. Immunopharmacol..

[11] Saran A., Nishizaki D., Lippman S.M., Kato S., Kurzrock R. (2025). Interleukin-17: A Pleiotropic Cytokine Implicated in Inflammatory, Infectious, and Malignant Disorders. Cytokine Growth Factor Rev..

[12] Furue M., Furue K., Tsuji G., Nakahara T. (2020). Interleukin-17a and Keratinocytes in Psoriasis. Int. J. Mol. Sci..

[13] Kong B., Lai Y. (2024). Il-17 Family Cytokines in Inflammatory or Autoimmune Skin Diseases. Adv. Immunol..

[14] Goepfert A., Barske C., Lehmann S., Wirth E., Willemsen J., Gudjonsson J.E., Ward N.L., Sarkar M.K., Hemmig R., Kolbinger F. (2022). Il-17-Induced Dimerization of Il-17ra Drives the Formation of the Il-17 Signalosome to Potentiate Signaling. Cell Rep..

[15] Raucci F., Saviano A., Casillo G.M., Guerra-Rodriguez M., Mansour A.A., Piccolo M., Ferraro M.G., Panza E., Vellecco V., Irace C. (2022). Il-17-Induced Inflammation Modulates the Mpges-1/Ppar-Γ Pathway in Monocytes/Macrophages. Br. J. Pharmacol..

[16] Liang J., Dai W., Liu C., Wen Y., Chen C., Xu Y., Huang S., Hou S., Li C., Chen Y. (2024). Gingerenone a Attenuates Ulcerative Colitis Via Targeting Il-17ra to Inhibit Inflammation and Restore Intestinal Barrier Function. Adv. Sci..

[17] Gong S., Xu R., Wang Y., Mao S., Zhang Y., Bu Q., Yang R., Wang T., Yang Y. (2025). Danggui Beimu Kushen Pill Alleviates Colitis-Induced Inflammation in Mice by Regulating the Il-6/Il-6r and Il-17a/Il-17ra Signaling Pathways. Pharmaceuticals.

[18] Li X., Qi T., Zhou L., Lin P., Chen Q., Li X., He R., Yang S., Liu Y., Qi F. (2025). Isoliquiritigenin Alleviates Abnormal Sarcomere Contraction and Inflammation in Myofascial Trigger Points Via the Il-17ra/Act1/P38 Pathway in Rats. Phytomedicine.

[19] Li Y.H., Zou M., Han Q., Deng L.R., Weinshilboum R.M. (2020). Therapeutic Potential of Triterpenoid Saponin Anemoside B4 from Pulsatilla Chinensis. Pharmacol. Res..

[20] Zhong J., Tan L., Chen M., He C. (2022). Pharmacological Activities and Molecular Mechanisms of Pulsatilla Saponins. Chin. Med..

[21] Liang Q.H., Li Q.R., Chen Z., Lv L.J., Lin Y., Jiang H.L., Wang K.X., Xiao M.Y., Kang N.X., Tu P.F. (2024). Anemoside B4, a New Pyruvate Carboxylase Inhibitor, Alleviates Colitis by Reprogramming Macrophage Function. Inflamm. Res..

[22] Lv L., Li Q., Wang K., Zhao J., Deng K., Zhang R., Chen Z., Khan I.A., Gui C., Feng S. (2024). Discovery of a New Anti-Inflammatory Agent from Anemoside B4 Derivatives and Its Therapeutic Effect on Colitis by Targeting Pyruvate Carboxylase. J. Med. Chem..

[23] Zhang Y., Zha Z., Shen W., Li D., Kang N., Chen Z., Liu Y., Xu G., Xu Q. (2021). Anemoside B4 Ameliorates Tnbs-Induced Colitis through S100a9/Mapk/Nf-Κb Signaling Pathway. Chin. Med..

[24] Ma H., Zhou M., Duan W., Chen L., Wang L., Liu P. (2020). Anemoside B4 Prevents Acute Ulcerative Colitis through Inhibiting of Tlr4/Nf-Κb/Mapk Signaling Pathway. Int. Immunopharmacol..

[25] Han Q., Deng L.R., Zou M., Tang H.Z., Huang C.Y., Chen F.J., Tomlinson B., Li Y.H. (2022). Anemoside B4 Protects against Chronic Relapsing Colitis in Mice by Modulating Inflammatory Response, Colonic Transcriptome and the Gut Microbiota. Phytomedicine.

[26] Luo D., Yan L., Wang Z., Ji X., Pei N., Jia J., Luo Y., Ouyang H., Yang S., Feng Y. (2024). Pulchinenoside B4 Ameliorates Oral Ulcers in Rats by Modulating Gut Microbiota and Metabolites. Appl. Microbiol. Biotechnol..

[27] Yuan R., He J., Huang L., Du L.J., Gao H., Xu Q., Yang S. (2020). Anemoside B4 Protects against Acute Lung Injury by Attenuating Inflammation through Blocking Nlrp3 Inflammasome Activation and Tlr4 Dimerization. J. Immunol. Res..

[28] Zhao C., Hu X., Bao L., Wu K., Feng L., Qiu M., Hao H., Fu Y., Zhang N. (2021). Aryl hydrocarbon receptor activation by Lactobacillus reuteri tryptophan metabolism alleviates Escherichia coli-induced mastitis in mice. PLoS Pathog..

[29] Lopes T.S., Fontoura P.S., Oliveira A., Rizzo F.A., Silveira S., Streck A.F. (2020). Use of Plant Extracts and Essential Oils in the Control of Bovine Mastitis. Res. Vet. Sci..

[30] Beccaria C., Baravalle C., Silvestrini P., Renna M.S., Molineri A.I., Signorini M.L., Neder V.E., Archilla G.A.S., Calvinho L.F., Dallard B.E. (2021). Efficacy of Panax Ginseng Extract Combined with Cephalexin as a Dry Cow Therapy. J. Dairy Res..

[31] Danev N., Harman R.M., Sipka A.S., Oliveira L., Huntimer L., Van De Walle G.R. (2025). The Secretomes of Bovine Mammary Epithelial Cell Subpopulations Differentially Modulate Macrophage Function. Vet. Q..

[32] Wellnitz O., Bruckmaier R.M. (2021). Invited Review: The Role of the Blood-Milk Barrier and Its Manipulation for the Efficacy of the Mammary Immune Response and Milk Production. J. Dairy Sci..

[33] Li K., Hou M., Zhang L., Tian M., Yang M., Jia L., Liang Y., Zou D., Liu R., Ma Y. (2022). Analysis of Antimicrobial Resistance and Genetic Correlations of Escherichia Coli in Dairy Cow Mastitis. J. Vet. Res..

[34] Orsi H., Guimarães F.F., Leite D.S., Guerra S.T., Joaquim S.F., Pantoja J.C.F., Hernandes R.T., Lucheis S.B., Ribeiro M.G., Langoni H. (2023). Characterization of Mammary Pathogenic Escherichia Coli Reveals the Diversity of Escherichia Coli Isolates Associated with Bovine Clinical Mastitis in Brazil. J. Dairy Sci..

[35] Gross J.J., Schwinn A.C., Bruckmaier R.M. (2021). Free and Bound Cortisol, Corticosterone, and Metabolic Adaptations During the Early Inflammatory Response to an Intramammary Lipopolysaccharide Challenge in Dairy Cows. Domest. Anim. Endocrinol..

[36] Zaatout N. (2022). An Overview on Mastitis-Associated Escherichia Coli: Pathogenicity, Host Immunity and the Use of Alternative Therapies. Microbiol. Res..

[37] Liu C., Tang X., Zhang W., Li G., Chen Y., Guo A., Hu C. (2019). 6-Bromoindirubin-3′-Oxime Suppresses Lps-Induced Inflammation Via Inhibition of the Tlr4/Nf-Κb and Tlr4/Mapk Signaling Pathways. Inflammation.

[38] Wang Z., Qin M., Dou Y., Hu X., Zhou R., Li Y., Wang J., Luoreng Z., Wang X. (2025). Circ_Homer3 Exacerbates Inflammatory Injury in Bovine Mammary Epithelial Cells Via Tnf/Mapk9 Activation and Ncoa4-Mediated Ferroptosis. Int. J. Biol. Macromol..

[39] Zhang H., Shao D., Yang Z., Wang S., Zhang Y., Peng H., Su Z., Zhang Y. (2025). Efficacy of Pulsatilla Saponin B4 for Treatment Dairy Cows Affected with Clinical Mastitis. PLoS ONE.

[40] Huangfu L., Li R., Huang Y., Wang S. (2023). The Il-17 Family in Diseases: From Bench to Bedside. Signal Transduct. Target. Ther..

[41] Rex D.a.B., Dagamajalu S., Gouda M.M., Suchitha G.P., Chanderasekaran J., Raju R., Prasad T.S.K., Bhandary Y.P. (2023). A Comprehensive Network Map of Il-17a Signaling Pathway. J. Cell Commun. Signal..

[42] Zhao L., Jin L., Yang B. (2023). Saikosaponin A Alleviates Staphylococcus Aureus-Induced Mastitis in Mice by Inhibiting Ferroptosis Via SIRT1/Nrf2 Pathway. J. Cell. Mol. Med..

[43] Wang H., Chen C., Chen X., Zhang J., Liu Y., Li X. (2021). Pk/Pd Modeling to Assess Rifaximin Clinical Dosage in a Mouse Model of Staphylococcus Aureus-Induced Mastitis. Front. Vet. Sci..

[44] Wang D., Wei Y., Shi L., Khan M.Z., Fan L., Wang Y., Yu Y. (2020). Genome-Wide DNA Methylation Pattern in a Mouse Model Reveals Two Novel Genes Associated with Staphylococcus Aureus Mastitis. Asian-Australas. J. Anim. Sci..

[45] Zheng X., Yang N., Mao R., Hao Y., Teng D., Wang J. (2024). Pharmacokinetics and Pharmacodynamics of Antibacterial Peptide Nzx in Staphylococcus Aureus Mastitis Mouse Model. Appl. Microbiol. Biotechnol..

[46] Tong C., Chen T., Chen Z., Wang H., Wang X., Liu F., Dai H., Wang X., Li X. (2021). Forsythiaside a Plays an Anti-Inflammatory Role in Lps-Induced Mastitis in a Mouse Model by Modulating the Mapk and Nf-Κb Signaling Pathways. Res. Vet. Sci..

[47] Gao Y., Hao Z., Zhang H., Liu J., Zhou G., Wen H., Su Q., Tong C., Huang S., Wang X. (2024). Forsythiaside a Attenuates Lipopolysaccharide-Induced Mouse Mastitis by Activating Autophagy and Regulating Gut Microbiota and Metabolism. Chem.-Biol. Interact..

[48] Schneider P., Salamon H., Weizmann N., Nissim-Eliraz E., Lysnyansky I., Shpigel N.Y. (2023). Immune Profiling of Experimental Murine Mastitis Reveals Conserved Response to Mammary Pathogenic Escherichia coli, Mycoplasma bovis, and Streptococcus uberis. Front. Microbiol..

[49] Li T., Fan Q.Y., Li X.M., Liu W.W., Xie F., Wang X.M., Dai X.F. (2022). Research Progress on Establishment of Mouse Mastitis Model and Prevention and Treatment with Chinese Herbal Medicine. Chin. J. Anim. Sci..

