# Supplementary figures and images for: Pulsatilla Saponin B4 Ameliorates LPS-Induced Inflammatory Response by Inhibiting IL-17RA and MAPK/NF-κB Signaling in Bovine Mammary Epithelial Cells and Mastitis Mouse Model
Source: Vet Sci. 2026 May 27;13(6):521. doi: 10.3390/vetsci13060521 (PMC13307605; doi:10.3390/vetsci13060521)

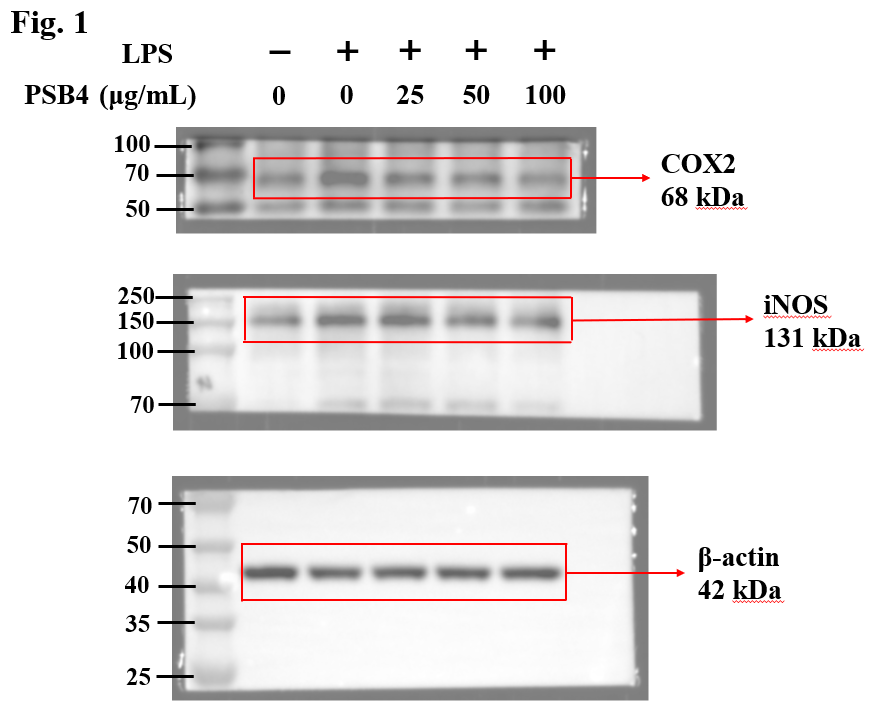

Supplement: Supplementary file 1 [file vetsci-13-00521-s001.zip › Figure S1.tif]

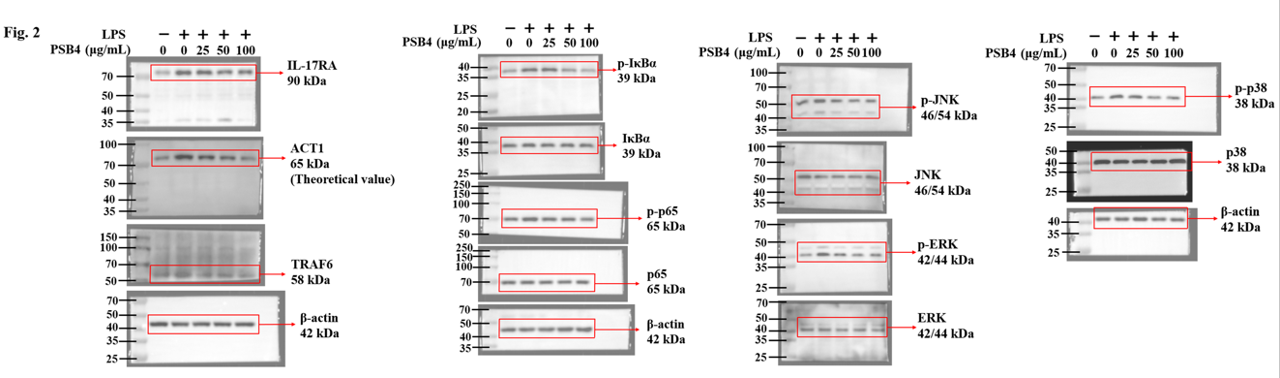

Supplement: Supplementary file 1 [file vetsci-13-00521-s001.zip › Figure S2.tif]

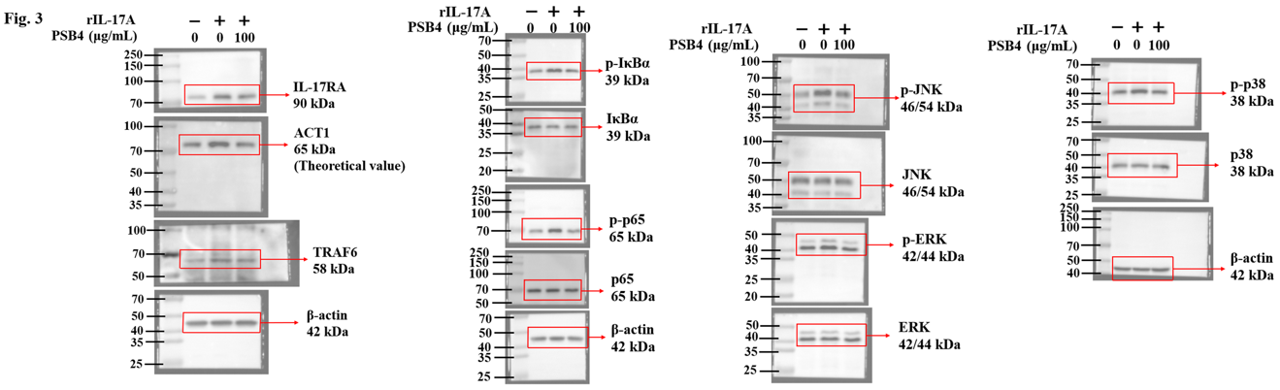

Supplement: Supplementary file 1 [file vetsci-13-00521-s001.zip › Figure S3.tif]

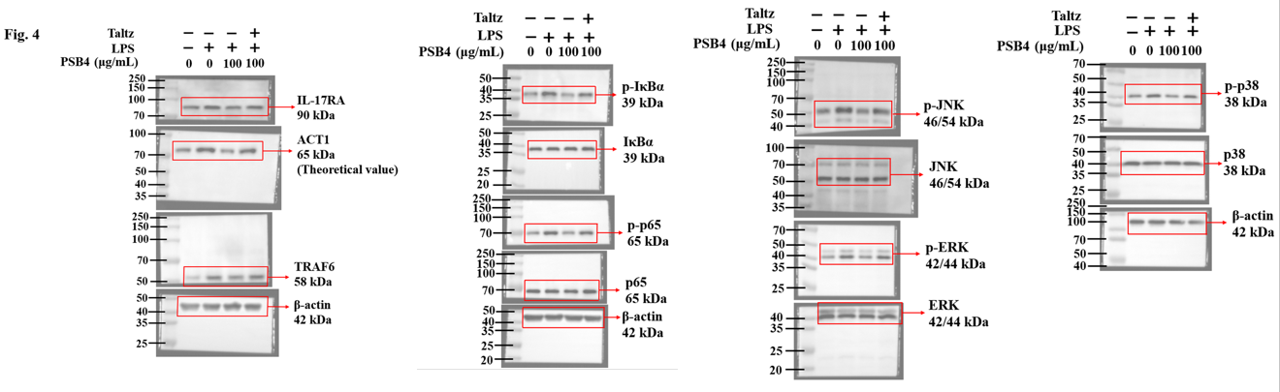

Supplement: Supplementary file 1 [file vetsci-13-00521-s001.zip › Figure S4.tif]

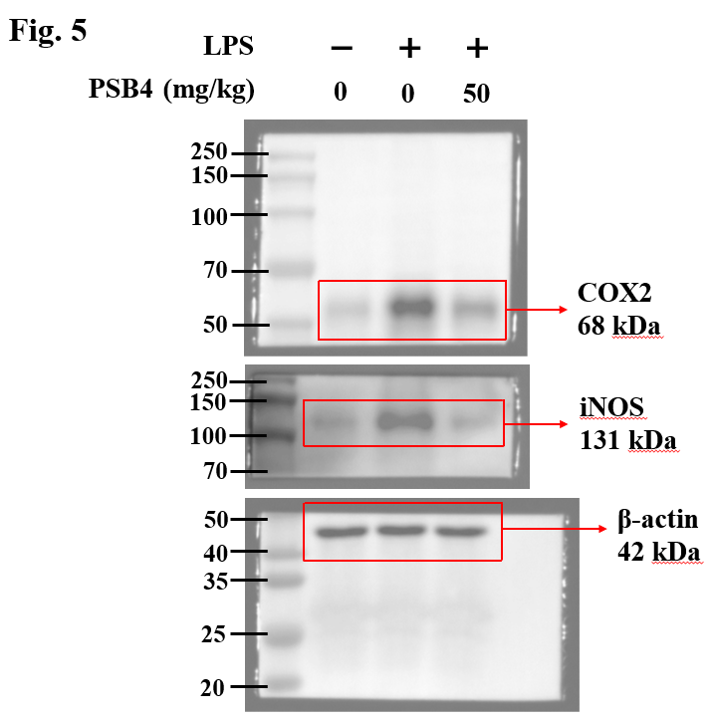

Supplement: Supplementary file 1 [file vetsci-13-00521-s001.zip › Figure S5.tif]

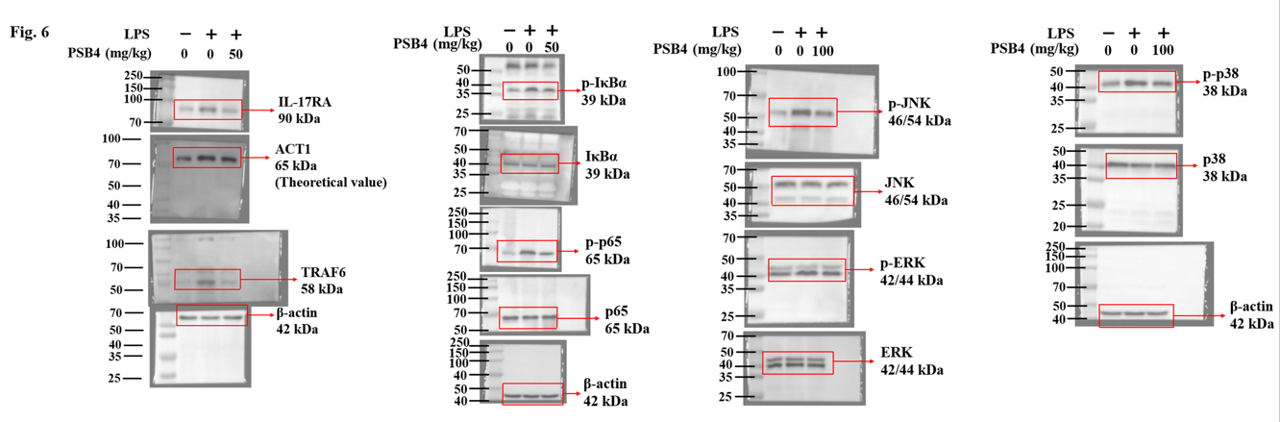

Supplement: Supplementary file 1 [file vetsci-13-00521-s001.zip › Figure S6.tif]
